# Episodic Future Thinking in Autism Spectrum Disorder and 22q11.2 Deletion Syndrome: Association with Anticipatory Pleasure and Social Functioning

**DOI:** 10.1007/s10803-021-04903-2

**Published:** 2021-02-14

**Authors:** Clémence Feller, Charlotte Dubois, Stephan Eliez, Maude Schneider

**Affiliations:** 1 Clinical Psychology Unit for Intellectual and Developmental Disabilities, Faculty of Psychology and Educational Sciences, University of Geneva, 40, Boulevard du Pont-d’Arve, 1205 Geneva, Switzerland; 2 Developmental Imaging and Psychopathology Lab Research Unit, Faculty of Medicine, University of Geneva, Geneva, Switzerland; 3 Department of Genetic Medicine and Development, Faculty of Medicine, University of Geneva, Geneva, Switzerland; 4 Department of Neurosciences, Center for Contextual Psychiatry, KU Leuven, Leuven, Belgium

**Keywords:** Episodic future thinking, Autism spectrum disorder, 22q11.2 deletion syndrome, Anticipatory pleasure, Social functioning, Autonoetic consciousness

## Abstract

Episodic future thinking (EFT) has been suggested to underlie anticipatory pleasure (AP), itself known to play a crucial role in social functioning (SF). Both AP and SF are impaired in various clinical populations, including autism spectrum disorders (ASD) and 22q11.2 deletion syndrome (22q11DS). Therefore, the relationship between EFT, AP and SF was investigated, as well as the potential role of projecting oneself in a social vs. non-social context. Seventy-seven participants [24 with 22q11DS, 20 with ASD, 33 typically developing controls (TDs)] (aged 12–25) were included. They were assessed with a future thinking task in which they were asked to recall a memory and produce a likely event. Narratives were rated based of specificity, richness and imaginability. Participants completed questionnaires assessing AP and SF. Narratives from ASD and 22q11DS participants were rated as less vivid compared to TDs. However, the characteristics of the narratives differed between ASD and 22q11DS participants in terms of specificity and level of details, as well as in reaction to social condition. Moreover, correlations were found between AP and EFT in both ASD and 22q11DS participants, and between SF and EFT in ASD participants. These results point towards impairments in EFT in both ASD and 22q11DS participants but with a specific profile in each condition. The observed associations between EFT and AP suggest that decreased autonoetic consciousness might underlie AP impairments. In ASD individuals, the association between SF and EFT highlights the need to better characterize EFT since EFT could be another mechanism contributing to social difficulties.

**Supplementary Information:**

The online version contains supplementary material available at 10.1007/s10803-021-04903-2.

## Introduction

Episodic future thinking (EFT) has been described as the ability to pre-experience future events, in other words to mentally project oneself into the future. It is the future equivalent of the episodic aspect of autobiographical memory, which consists of remembering or re-experiencing past events (Atance and O’Neill 2001). Both episodic memory (EM) and EFT require autonoetic consciousness, defined as the ability to maintain self-continuity by projecting oneself into the past, present or future (Gardiner 2001; Klein 2016), since self-projection is a core mechanism of both EM and EFT (Suddendorf and Corballis 1997). It also requires mentally travelling through time in order to reexperience or pre-experience an event (Suddendorf and Corballis 1997). EM and EFT appear to be relevant for social functioning (SF)—described as the ability to form and maintain social relationships (Campbell et al. 2015)—since individuals’ self-awareness is suggested to play a role in social behavior (Gardiner 2001). EM and EFT are thought to mature simultaneously in typical development (Suddendorf 2010), emerging around 4 years of age (Atance and O’Neill 2005), and both abilities decline in older adults (Addis et al. 2011). Impairments in EFT have been reported in several populations experiencing mental health issues [for a systematic review see (Hallford et al. 2018)] but EFT has been more rarely examined in neurodevelopmental disorders, despite the fact that SF impairments are frequently observed in this population.

It has been suggested that EFT is one of the cognitive functions underlying anticipatory pleasure [AP; i.e. pleasure related to future activities, (Gard et al. 2007)], since individuals need to activate representations of future experiences—in other words to project themselves in the future—in order to anticipate the potential pleasure associated with these experiences (Kring and Caponigro 2010). This has been partially supported by several studies observing fewer anticipated positive future events (Bjärehed et al. 2010; Hallford and Sharma 2019; MacLeod and Salaminiou 2001) and lower AP (Wu et al. 2017) in participants with depression, but without examining EFT and AP conjointly. However, a recent study observed a direct link between these two constructs, showing that individuals with major depression presented lower AP and projected themselves in the future with less specificity and less vividness, as well as with less associated pleasure (Hallford et al. 2020a). Even if AP can involve a large range of experiences, impaired hedonic capacity for interpersonal or social experiences (interpersonal AP) is particularly relevant in the field of psychology (for a review see Gooding and Madison 2019), as it plays a critical role in SF (e.g., Buck and Lysaker 2013; Granholm et al. 2013; Moore et al. 2019; Ritsner et al. 2018). Therefore, it appears of crucial importance to examine the potential association between EFT in a social context (i.e. projecting oneself in the future in the company of at least another person) and interpersonal AP. In the present study, we examined the associations between EFT in social vs. non-social contexts, interpersonal AP and SF in two neurodevelopmental disorders characterized by pronounced social impairments (Fakhoury 2015; Norkett et al. 2017; Schneider et al. 2012; Schonherz et al. 2014; Seltzer et al. 2004; Shashi et al. 2012; Stoddard et al. 2010; Wallace et al. 2017; Yang et al. 2016) and impaired AP (Dubourg et al. 2017; Han et al. 2019; Novacek et al. 2016): 22q11.2 deletion syndrome (22q11DS) and autism spectrum disorders (ASD). Even if these two populations are both characterized by social impairments, our goal is to explore to what extent the psychological processes underlying social impairments overlap in these two groups or show high levels of specificity.

22q11DS is a neurogenetic condition affecting 1:2000–4000 live births and is one of the highest risk factors for developing schizophrenia spectrum disorders (Schneider et al. 2014). Cognitively, individuals with 22q11DS present a normally distributed IQ but in the low range (mean IQ = 70), and are characterized by impairments in verbal initiation (Maeder et al. 2016). The syndrome is associated with a heterogenous phenotype of behavioral and clinical characteristics, including impairments in SF [i.e. lower adaptive behavior skills (Schneider et al. 2014)] and social skills (Norkett et al. 2017; Shashi et al. 2012). Some authors have suggested that a significant proportion of 22q11DS meet criteria for ASD (e.g., Vorstman et al. 2006), but other findings have highlighted differences in the social phenotype between 22q11DS and idiopathic ASD (Angkustsiri et al. 2014). Participants with 22q11DS also frequently exhibit negative symptoms of psychosis, such as social withdrawal, anhedonia and amotivation (Schneider et al. 2012; Schonherz et al. 2014; Stoddard et al. 2010) that lead to poor outcomes (Schneider et al. 2014). Of interest, a recent study of Dubourg et al. (2017) found deficits in AP in 22q11DS individuals that was related to the severity of negative symptoms, but the link with social impairments was not tested. Finally, to the best of our knowledge, no study has explored EFT abilities in 22q11DS individuals, nor the autobiographical component of EM.

ASD is a neurodevelopmental disorder affecting 1:54 individuals and is characterized by alterations in social communication and interactions, leading to social impairments (Fakhoury 2015; Maenner et al. 2020), as well as by repetitive behaviors and restricted interests. Reduced social interactions (i.e. social withdrawal) appear very early on (Seltzer et al. 2004; Wallace et al. 2017) and can lead to lower social participation and higher isolation in adulthood (Orsmond et al. 2013). Moreover, individuals with ASD are impaired in SF [i.e. lower adaptive behavior skills in terms of socialization (Pugliese et al. 2015; Yang et al. 2016)]. In addition, decreased AP—and interpersonal AP in particular—has been observed (Novacek et al. 2016), and the intensity of this decrease appears to be similar to that of adults with clinical depression (Han et al. 2019). Finally, it has been well established that individuals with ASD show deficits in autobiographical EM (Crane and Goddard 2008), and there is now a growing literature showing impairments in EFT as well. Several studies found impairments in EFT in adults in terms of narratives’ specificity (Lind et al. 2014; Lind and Bowler 2010) as well as difficulties in self-projection and scene-construction (Lind et al. 2014). However, Crane et al. (2013) did not find the same results but used a methodology of sentence completion that appeared to be insensitive to capture EFT (Lind and Williams 2012). Studies focusing on children and adolescents also found greater difficulties in generating scenarios happening in the future (Jackson and Atance 2008; Terrett et al. 2013). Less vivid narratives (Anger et al. 2019) along with difficulties in scene-construction (Ciaramelli et al. 2018), self-projection (Hanson and Atance 2014; Marini et al. 2016) and narrative skills (Marini et al. 2019) were also reported. However, the link between EFT, AP and SF impairments has, to our knowledge, never been studied.

### Aims

**Fig. 1:**
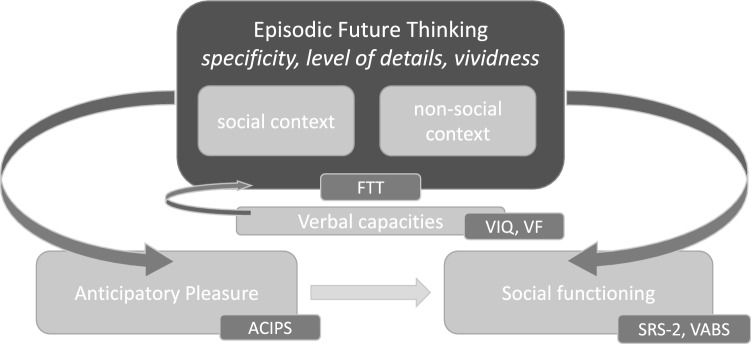
Relationships between EFT, AP and SRS

In the present study, we aimed to investigate the characteristics of EFT in adolescents and young adults with 22q11DS and ASD. (1) Our first hypothesis was that both groups would show EFT impairments, inducing less specific, less detailed and less vivid narratives than TDs on a Future Thinking Task (FTT) in the production condition. (2) Secondly, we aimed to explore whether a social versus a non-social context would lead to different answers in FTT, with the hypothesis that the social context would be more challenging for individuals with 22q11DS and ASD since both groups are characterized by social impairments and social withdrawal. (3) Finally, we expected to find associations between EFT, interpersonal AP and SF in both individuals with 22q11DS and ASD. In particular, we expected to observe stronger associations between EFT and interpersonal AP or SF when EFT involved a social context. (4) Additionally, the potential impact of verbal capacities on EFT, as measured by verbal IQ and verbal initiation, was examined in the two groups (Fig. 1).

## Method

### Sample

Seventy-seven participants (39% female) aged between 12 and 25 were included in the study (mean age = 18.4, *SD* = 3.93). Twenty-four (33.3% female) were 22q11DS carriers (mean age = 18.09, *SD* = 4.02) and were recruited through the 22q11DS Swiss longitudinal cohort. Twenty (35% female) were diagnosed with ASD (mean age = 17.43, *SD* = 4.13) and were recruited through announcements to parent associations in Switzerland and France and specialized clinical centers in Geneva and France. Thirty-three (45% female,) were part of the TD group (mean age = 18.60, *SD* = 3.80) and were recruited through the siblings of the 22q11DS Swiss longitudinal cohort and from announcements at the University of Geneva. Participants were not statistically different in terms of age and gender but differed on full-scale IQ scores (Table 1). This study was approved by the Swiss Ethics Committees on research involving humans (Commission Cantonale d’Ethique de la Recherche sur l’Etre Humain—CCER) of Geneva (CH). Parents had to give their written consent for all participants with ASD and 22q11DS, regardless of their age, as well as for TDs under 18 years.

**Table 1:** Participant characteristics, psychiatric diagnosis and psychotropic medication

	Diagnostic group			Comparison					
				TDs-22q11DS		TDs-ASD		22q11DS-ASD	
	TDs	22q11DS	ASD	Statistical test	*p* value	Statistical test	*p* value	Statistical test	*p* value
*N*	33	24	20						
Gender [female (%)]	15 (45%)	8 (50%)	7 (35%)	*χ*2 = 0.848	0.357	χ2 = 0.561	0.454	*χ*2 = 0.013	0.908
Age [mean (SD)]	18.60 (3.80)	18.09 (4.02)	17.43 (4.13)	*t* = 0.490	0.626	*t* = 1.050	0.299	*t* = 0.533	0.597
Full scale IQ [mean (SD)]	109.16 (13.22)	73.00 (16.49)	101.75 (19.47)	*t* = 8.906	**0.000**	*t* = 1.615	0.113	*t* = -5.242	**0.000**
Psychiatric diagnosis [*N *(%)]									
Simple phobia		5 (20.83%)	3 (15%)						
Agoraphobia		0	2 (10%)						
Social phobia		1 (4.16%)	4 (20%)						
Generalized anxiety		5 (20.83%)	1 (5%)						
Attention deficit disorder		6 (25%)	4 (20%)						
Persistant depressive disorder		0	2 (10%)						
Major depressive episode		0	2 (10%)						
Psychosis		0	0						
Obsessive–compulsive disorder		0	0						
ADOS-2 module 3 (*N* = 7) (mean)									
ADOS-2 total score			11.43						
ADOS-2 SA score			8.28						
ADOS-2 RRB score			3.14						
ADOS-2 module 4 (*N* = 13) (mean)									
ADOS-2 total score			12.85						
ADOS-2 SA score			9.38						
ADOS-2 RRB score			3.46						
ADI-R (*N* = 9) (mean)									
ADI-R domain A			18						
ADI-R domain B			13.33						
ADI-R domain C			5.77						
SCQ (*N* = 70) (mean)									
SCQ total score	3.07	8.87	18.77						
Psychotropic medication									
Total [*N*(%)]		12 (50%)	6 (30%)						
Categories									
Psychostimulant		8 (33.33%)	2 (10%)						
Antidepressants		4 (16.66%)	1 (5%)						
Neuroleptics		4 (16.66%)	2 (10%)						
Antiepilecptics		2 (8.33%)	0						
Anxiolytics		2 (8.33%)	1 (5%)						

Significant p-values at the 0.05 level are displayed in bold

TDs had been screened for psychiatric diagnostics conforming to our exclusion criterias

Inclusion criteria for all participants were (1) age between 12 and 25 years, (2) sufficient command of the French language, (3) sufficient verbal and cognitive capacities (intellectual deficiency was not an exclusion criterion, participants who showed satisfying comprehension abilities to perform the task were included). All participants from the 22q11DS group had a confirmed genetic diagnosis of microdeletion 22q11.2. They were screened with the Social Communication Questionnaire (SCQ; Rutter et al. 2003a) with a mean score of 8.87. Five participants with 22q11DS had a score above the clinical cutoff (15). All participants from the ASD group had a confirmed clinical diagnosis of ASD and completed the Autism Diagnostic Observation Schedule second version (ADOS-2; Lord et al. 2012). Principal caregivers completed either the Autism Diagnostic Interview-Revised (ADI-R; Rutter et al. 2003b) or the SCQ. Participants with 22q11DS and ASD were screened for comorbid psychiatric disorders using validated semi-structured instruments: Diagnostic Interview for Children and Adolescents-Revised (DICA; Reich 2000) or Schedule for Affective Disorders and Schizophrenia for School-Age Children Present and Lifetime Version (K-SADS-PL DSM-5; Kaufmann et al. 2016) for participants under 18 years old and Structured Clinical Interview for DSM-IV Axis I (SCID-I; Williams 1996) or DSM-V (SCID-5-CV; First et al. 2016) for participants above 18 years old (Table 1). Exclusion criteria for participants included in TDs were (1) being born preterm, (2) having a first degree relative diagnosed with a developmental disorder (siblings of participants with 22q11DS were included if the 22q11.2 deletion was confirmed to be de novo), (3) having a lifetime history of psychiatric (including neurodevelopmental disorders such as ASD), neurologic, or learning impairments. Of note, TDs were screened using the SCQ, with a mean score of 3.07 and none of the participants being above the clinical cutoff.

### Material

#### Future Thinking Task (FTT) in Social and Non-social Contexts

EFT was assessed using an adaptation of a previous task (D’Argembeau et al. 2008). Participants had to *recall* personal past events (i.e. recollection condition) and to *imagine* plausible future events that could likely happen to them (i.e. production condition). For both temporal conditions (recollection and production), the instructions were to generate specific events (i.e. events that took place in a specific location on a specific day and lasted no longer than a day) based on a cue word (school/work, home, holidays, weekend). Based on other studies investigating EFT (e.g., Lind et al. 2014), participants were specifically instructed to give as many details as possible, including olfactive/gustative, visual and auditive details but also thoughts, feelings and actions. In order to specifically investigate EFT in social vs. non-social contexts, participants were asked to tell narratives related to a *social* (in which they were at least with one person) and *non-social* (in which they were alone) condition for each word. In total, participants had to tell 16 narratives [i.e. four for each word, one narrative per condition (temporal: recollection and production; social: non-social and social)]. The task was designed on E-Prime Software. Instructions appeared on the screen. Participants were given a maximum of 90 s to start the narrative, and a maximum of 90 s to produce the narrative. The order of the words and conditions for each word was randomized. All responses were audiotaped for scoring purposes.

All the narratives (n = 1232) were initially reviewed to check if participants respected the given conditions (i.e. recollection vs production, non-social vs social, narrative related to the target word). Narratives that did not fit a condition as well as missing narratives were excluded (N = 167; 22q11DS n = 90, ASD n = 62, TD n = 15). The scoring was divided into three parts. First, each narrative was classified in different categories, as described in D’Argembeau et al. (2008): (1) *specific*: events that took place in a specific location on a specific day, and lasted no longer than a day; (2) *extended*: events lasting more than a day; (3) *categoric*: collection of events that were not related to one another or did not refer to a specific period of time. Secondly, an “*experiential index*” (e.g., Lind et al. 2014) was computed by doing the sum of each sub-categories of details (olfactive/gustative, visual, auditive, thoughts, feelings and actions) given by the participants. Each of these six sub-categories was rated from 0 (no detail) to 2 (2 or more details). This index indicates the narratives level of details, its richness. Finally, a “*subjective appreciation index*” (rated from 1 = not at all to 5 = extremely) was given for each narrative, reflecting how vivid the interviewer subjectively perceived the narrative. This index indicates the narratives vividness, its imaginability. The final scores included in the analyses were calculated as followed: (1) *narratives categories*: proportion of specific, extended and categoric narratives for each condition (temporal and social), (2) *experiential index*: sum of each sub-categories of details for all narratives in each condition, (3) *subjective appreciation index*: sum of each subjective appreciation scores in each condition, 4) *score of difference for both experiential index and subjective appreciation index*: in order to take into account episodic memory impairments and to extract a “purer” measure of EFT, the recollection score was subtracted from the production score. Three different examiners (CF, MS, CD) administered the task and double-scored the narratives of a subsample of 42 (55%) participants (at least 50% per group). When the score differed between the two raters for the subjective appreciation index, the mean score was computed. See Supplementary Material for a narrative example and its scoring.

#### Verbal Capacities

Subjective intellectual functioning was assessed using children or adults Wechsler intelligence scales (WISC-III, Wechsler 1991), (WISC-IV, Wechsler 2004), (WISC-V, Wechsler 2014), (WAIS-III, Wechsler 1997), (WAIS-IV, Wechsler 2011). Only Verbal IQ score was used in the current study.

The semantic verbal fluency test (animal category) was also administered to assess verbal initiation.

#### Adaptive Functioning

The Vineland Adaptive Behaviour Scale, 2nd Edition (VABS-II, Sparrow et al. 2015) was administered to parents whose teenagers were still living at home (N = 63; TD = 23, ASD = 17, 22q11DS = 23) to assess adaptive functioning. Only the socialization dimension was used in the analyses using appropriate standardized scores (M = 100; SD = 15). The rationale was to have a pure measure of social functioning that the total score would have failed to capture since the VABS is made of two other domains, communication and daily-life skills, that are not related to our topic of interest.

#### Questionnaires

All participants completed the French version of the Anticipatory and Consummatory Interpersonal Pleasure Scale (ACIPS; Gooding and Pflum 2014), which measures both anticipatory and consummatory dimensions in a social context. Parents of all participants completed a questionnaire about demographic and medical characteristics, as well as the Social Responsiveness Scale (SRS-2; Constantino 2012), School-Age version (< 18 years old) or Adult parent-report version (> 18 years old), that measures deficits in social behavior. Only the Social Communication and Interaction score was used for analyses, for the same reason than the one explained in the previous section.

### Statistical Analyses

Statistical analyses were conducted with IBM SPSS Statistics 26. As the distribution of our variables of interest did not follow a normal distribution (Shapiro–Wilk tests,* p* < 0.05), between-group comparisons and correlations were performed with non-parametric statistics (Kruskal–Wallis tests and Spearman correlations). For post-hoc analyses, only adjusted* p* values are reported to consider Bonferroni correction for multiple tests. For correlations, the Benjamini-Hochberg (BH) multiple comparison correction was applied and therefore only* p* values that survived BH comparisons are reported. First, groups comparisons were conducted regarding the narratives’ categories (% of specific, extended and categoric narratives) in all conditions. Secondly, the mean experiential index and mean subjective appreciation index were compared across the three groups in all the conditions. We chose not to use IQ as a covariate as lower IQ is part of the neurodevelopmental disorders phenotype (Dennis et al. 2009), and therefore covariating for IQ would remove some of the variance inherent in the diagnosis. However, we examined the link between the FTT and verbal capacities in each group independently as described: spearman correlations were used to examine the associations between FTT and verbal initiation [i.e. verbal fluency animal category (VF)] as well as verbal cognitive abilities [i.e. verbal IQ (VIQ)]. Finally, in order to specifically examine EFT, the scores of differences were compared in term of experiential index and subjective appreciation index between the three groups. Spearman correlations were used to examine the association between EFT variables and AP (i.e. ACIPS) as well as SF (i.e. SRS-2 and VABS-II).

## Results

### Narratives’ Categories: Narratives’ Specificity

*Overall* the proportion of *specific*, *extended* and *categoric* narratives in the three groups across the different categories (social and temporal conditions) is displayed in Table 2, as well as statistical values. No statistically significant differences were observed between the three groups regarding the proportion of *specific* and *extended* answers. However, significant differences emerged across groups regarding the proportion of *categoric* answers. Specifically, TDs produced significantly less categoric narratives compared to both participants with 22q11DS and ASD. The two neurodevelopmental groups were not statistically different from each other.

**Table 2:** Narratives’ categories

	Performance median % (IIQ)			Groups comparisons			Post-hoc analyses								
							TDs-22q11DS			TDs-ASD			22q11DS-ASD		
	TDs	22q11DS	ASD	Kruskal–Wallis test	*p* value	η^2^	Test statistic	*p* value	Adjusted *p* value	Test statistic	*p* value	Adjusted *p* value	Test statistic	*p* value	Adjusted *p* value
Overall performance															
Specific	75.00 (31.25)	59.82 (44.72)	51.67 (49.18)	5.271	.072										
Extended	13.33 (13.75)	13.33 (12.19)	6.46 (16.07)	2.088	.352										
Categoric	7.14 (20.00)	20.09 (45.00)	28.57 (38.47)	12.577	**.002**	0.153	− 14.314	.016	**0.49**	− 21.089	.001	**.002**	− 6.775	.314	.941
Performance according to the temporal condition															
Specific															
Recollection	87.50 (37.50)	69.05 (37.50)	61.25 (62.50)	6.895	**0.032**	0.066	13.252	.025	.075	13.381	.032	.096	.129	.985	1.000
Production	50.00 (42.86)	53.57 (68.93)	46.43 (40.63)	2.400	.301										
Extended															
Recollection	0.00 (12.50)	17.14 (25.89)	0.00 (17.50)	3.836	.147										
Production	25.00 (25.00)	0.00 (14.29)	6.25 (19.96)	10.348	**.006**	0.248	17.155	.003	**.009**	14.180	.020	.061	− 2.975	.648	1.000
Categoric															
Recollection	0.00 (12.50)	12.50 (25.00)	22.50 (42.41)	12.415	**.002**	0.141	− 12.610	.025	.075	− 20.102	.001	**.002**	− 7.492	.238	.713
Production	12.50 (28.57)	28.57 (67.71)	40.18 (59.38)	9.892	**.007**	0.197	13.492	.023	.068	− 18.109	.004	**.011**	− 4.617	.490	1.000
Performance according to the social condition															
Specific															
Social	75.00 (25.00)	56.25 (52.68)	57.14 (39.73)	6.951	**.031**	0.067	13.483	.024	.071	13.512	.032	.096	.029	.997	1.000
Non-social	75.00 (37.50)	62.50 (59.82)	46.43 (54.76)	3.788	.150										
Extended															
Social	12.5 0(25.00)	19.64 (23.96)	12.50 (25.00)	2.449	.294										
Non-social	14.29 (25.00)	0.00 (12.50)	0.00 (14.88)	6.688	**.035**	0.063	13.777	.013	**.039**	9.718	.098	.293	− 4.058	.517	1.000
Categoric															
Social	0.00 (25.00)	14.29 (50.00)	25.00 (33.93)	10.565	**.005**	0.116	− 12.634	.030	.090	− 18.964	.002	**.006**	− 6.329	.335	1.000
Non-social	12.50 (25.00)	15.48 (63.54)	42.86 (49.85)	9.266	**.010**	0.098	− 11.206	.057	.170	− 18.227	− 3	**.010**	− 7.021	.290	.869

Significant *p* values at the 0.05 level after Bonferonni correction for multiple tests are displayed in **bold**

*Temporal condition *(*recollection and production*) no statistically significant differences were observed between the three groups regarding the proportion of *specific* answers. Significant differences were observed in the *production* condition regarding the percentage of *extended* narratives. In particular, TDs produced more *extended* narratives in the *production* condition compared to participants with 22q11DS. Finally, significant differences appeared in both *recollection* and *production* conditions regarding the percentage of *categoric* narratives. Specifically, TDs produced less *categoric* narratives than participants with ASD in both *recollection* and *production* conditions.

*Social condition *(*social and non-social*) no statistically significant differences were observed between the three groups regarding the proportion of *specific* answers. Regarding the percentage of *extended* narratives, significant group differences emerged only in the *non-social* condition. Post-hoc analyses indicated that TDs produced statistically more *extended* narratives in the *non-social* condition than participants with 22q11DS. Finally, significant differences appeared in both *social* and *non-social* conditions regarding the percentage of *categoric* answers. In the two conditions, TDs produced statistically less *categoric* narratives than participants with ASD. See Table 2 for statistical values.

Of note, all the analyses were run without the five participants with 22q11DS that scored higher than the clinical cutoff on the SCQ and the results remained unchanged.

### Experiential Index: Level of Details/Richness

Statistically significant differences were found across groups in both social and temporal conditions (see Table 3 for statistical values). Specifically, post-hoc analyses indicated that participants with 22q11DS had a significantly lower mean experiential index (i.e. less detailed narratives) compared to TDs regardless of the condition, and also lower than participants with ASD in the social condition (both recollection and production). Contrary to our hypothesis, individuals with ASD had a significantly higher mean experiential index compared to TDs only in the non-social production condition. Additional analyses were conducted to subdivide the experiential index between sensory and non-sensory details, revealing more non-sensory details in TDs compared to ASD but no difference in sensory details (see Table 4), and note supplementary material. Of note, all the analyses were run without the five participants with 22q11DS that scored higher than the clinical cutoff at the SCQ and results remained unchanged.

**Table 3:** Experiential index and subjective appreciation performance

	Performance median (IIQ)			Groups comparisons			Post-hoc analyses								
							TDs-22q11DS			TDs-ASD			22q11DS-ASD		
	TDs	22q11DS	ASD	Kruskal–Wallis test	*p* value	η^2^	Test statistic	*p* value	Adjusted *p* value	Test statistic	*p* value	Adjusted *p* value	Test statistic	*p* value	Adjusted *p* value
Experiential index: total mean scores															
Recollection non-social	16.00 (6.00)	8.50 (6.00)	11.50 (8.50)	20.533	**.000**	0.25	27.123	.000	**.000**	12.602	.046	.139	− 14.521	.032	.095
Recollation social	17.00 (4.00)	9.50 (5.00)	15.00 (7.25)	25.139	**.000**	0.313	30.017	.000	**.000**	12.571	.047	.140	− 17.446	.010	**.029**
Production non-social	13.00 (7.00)	6.50 (5.00)	8.50 (9.25)	24.798	**.000**	0.308	29.337	.000	**.000**	17.587	.005	**.016**	− 11.750	.082	.247
Production social	14.00 (5.00)	6.00 (6.00)	11.50 (4.75)	25.253	**.000**	0.314	29.919	.000	**.000**	9.425	.136	.409	− 20.496	.002	**.007**
Recollection non-social	12.00 (4.00)	5.75 (4.00)	7.75 (6.00)	23.472	**.000**	0.29	27.513	.000	**.000**	20.534	.001	**.004**	− 6.979	.302	.906
Subjective appreciation: total mean scores															
Recollation social	13.00 (3.00)	8.25 (4.00)	9.25 (6.50)	29.374	**.000**	0.37	29.600	.000	**.000**	25.263	.000	**.000**	− 4.338	.520	1.000
Production non-social	10.50 (3.00)	4.50 (3.00)	6.75 (3.88)	30.083	**.000**	0.38	31.936	.000	**.000**	20.944	.001	**.003**	− 10.992	.104	.312
Production social	11.00 (3.50)	4.75 (3.00)	7.25 (4.75)	30.193	**.000**	0.381	32.313	.000	**.000**	19.617	.002	**.006**	− 12.696	.060	.181

Significant *p* values at the 0.05 level after Bonferonni correction for multiple tests are displayed in bold

**Table 4:** Experiential index sensory vs. non-sensory details

	Performance Median (IIQ)			Groups comparisons			Post-Hoc analyses					
							TDs-22q11DS		TDs-ASD		22q11DS-ASD	
	TDs	22q11DS	ASD	Kruskal–Wallis test	*p* value	η^2^	Test statistic	*p* value	Test statistic	*p* value	Test statistic	*p* value
Experiential index: total mean scores												
Sensory details overall	7 (7)	2 (6)	5 (9.25)	10.959	**.004**	0.121	10.401	**.001**	.045	.832	5.645	.018
Non-sensory details overall	54 (13)	27 (14)	14 (22.75)	29.217	**.000**	0.368	27.458	**.000**	8.101	**.004**	5.622	.018

Significant* p* values at the 0.05 level are displayed in bold

#### Association Between Experiential Index and Verbal Performance

*TD*: VIQ score correlated both with experiential index total scores in non-social recollection condition and in social production condition. No correlation was found with VF.

*22q11DS*: VF correlated with social production experiential index. No correlation was found with VIQ.

*ASD*: VIQ score correlated both with experiential index total scores in non-social and social recollection as well as in non-social production conditions. No correlation was found with VF. See Table 5 for statistical values.

**Table 5:** Correlation of experiential index and subjective appreciation performance with verbal performance

	TDs						22q11DS						ASD					
	Verbal IQ			Verbal fluency			Verbal IQ			Verbal fluency			Verbal IQ			Verbal fluency		
	Spearman correlation	Sig (2-tailed)	BH treshold	Spearman correlation	Sig (2-tailed)	BH treshold	Spearman correlation	Sig (2-tailed)	BH treshold	Spearman correlation	Sig (2-tailed)	BH treshold	Spearman correlation	Sig (2-tailed)	BH treshold	Spearman correlation	Sig (2-tailed)	BH treshold
Experiential index: total mean scores																		
Recollection non-social	.528	**.002**	.025	.341	.052	.016	.224	.303	.012	.115	.593	.006	.546	**.013**	.022	− .188	.427	.009
Recollation social	.355	.050	.019	.240	.178	.006	.192	.380	.009	.318	.130	.022	.627	**.003**	.025	.211	.371	.012
Production non-social	.274	.136	.012	.312	.233	.003	.298	.167	.019	.248	.242	.016	.532	**.016**	.019	.016	.946	.003
Production social	.458	**.010**	.022	.262	.141	.009	.060	.785	.003	.505	**.012**	.025	.384	.094	.016	.046	.848	.006
Subjective appreciation: total mean scores																		
Recollection non-social	.285	.121	.012	.279	.116	.016	.340	.112	.016	.176	.411	.006	.578	**.008**	.025	− .213	.367	.012
Recollation social	.311	.088	.019	.326	.065	.022	.226	.300	.009	.474	**.019**	.025	.553	**.011**	.019	− .174	.463	.009
Production non-social	.127	.498	.006	.092	.612	.003	.293	.174	.012	.376	.070	.019	.575	**.008**	.022	− .028	.905	.006
Production social	.345	.057	.025	.241	.178	.009	.161	.464	.003	.0469	**.021**	.022	.483	.031	.016	.000	.999	.003

Correlations sustaining Benjamini–Hochberg treshold are displayed in bold

Of note, all the analyses were run without the five participants with 22q11DS that scored higher than the clinical cutoff on the SCQ and the results remained unchanged.

### Subjective Appreciation Index: Imaginability/Vividness

Consistent findings appeared between all four conditions, with statistically significant differences across groups (see Table 3 for statistical values). More precisely, post-hoc analyses indicated that TDs had a higher subjective appreciation index (i.e. more vivid narratives) than both participants with 22q11DS and ASD across all temporal and social conditions. Of note, all the analyses were run without the five participants with 22q11DS that scored higher than the clinical cutoff at the SCQ and results remained unchanged.

#### Association Between Subjective Appreciation Index and Verbal Performance Among the Three Groups

*TD*: no correlation was found between VIQ nor VF and subjective appreciation scores.

*22q11DS*: VF correlated with subjective appreciation scores in social recollection and social production conditions. No correlation was found with VIQ.

*ASD*: VIQ score correlated with subjective appreciation scores in non-social recollection and production as well as in social recollection conditions. No correlation was found with VF. See Table 5 for statistical values.

Of note, all the analyses were run without the five participants with 22q11DS that scored higher than the clinical cutoff on the SCQ and the results remained unchanged.

### Episodic Future Thinking

To specifically investigate EFT in the three groups, the same analyses were run with the difference scores regarding experiential index (i.e. experiential index total score in recollection condition minus experiential index total score in production condition) and subjective appreciation index (i.e. subjective appreciation total score in recollection condition minus subjective appreciation total score in production condition). The group comparison revealed no statically significant difference across the different populations (see Table 6 for the results of the comparison in the social and non-social conditions).

**Table 6:** Future projection capacity: experiential index and subjective appreciation index

	Performances mean (SD)			Performance median (IIQ)			Groups comparisons	
	TDs	22q11DS	ASD	TDs	22q11DS	ASD	Kruskal–Wallis test	*p* value
Experiential index: difference scores								
Social	2.97 (3.25)	2.46 (3.53)	2.44 (4.48)	3.00 (5.00)	2.00 (3.00)	1.00 (6.50)	1.389	.499
Non-social	1.70 (4.22)	1.71 (3.62)	2.05 (5.07)	2.00 (4.00)	1.50 (4.00)	1.00 (7.50)	.136	.934
Overall	4.67 (6.02)	4.17 (5.48)	4.49 (6.62)	4.00 (4.00)	4.00 (6.00)	3.00 (8.25)	.766	.682
Subjective appreciation: difference scores								
Social	1.86 (2.24)	2.81 (2.02)	2.04 (2.34)	1.50 (3.00)	3.00 (3.00)	1.00 (4.25)	4.859	.088
Non-social	1.32 (2.38)	1.95 (5.50)	1.45 (2.32)	1.50 (2.50)	0.50 (2.50)	0.25 (3.25)	2.206	.332
Overall	3.18 (3.19)	4.75 (6.01)	3.49 (3.91)	3.00 (4.00)	3.50 (4.00)	2.25 (4.50)	2.808	.246

Significant *p* values at the 0.05 level after Bonferonni correction for multiple tests are displayed in bold

#### Association Between Experiential Index and Subjective Appreciation Index (Difference Scores) and Clinical Variables

*Anticipatory pleasure* no significant correlation was observed between the *experiential index total scores* and the ACIPS total score in any of the three groups, neither overall nor in the social/non-social conditions. The *subjective appreciation index* in the *social* condition was significantly associated with the ACIPS total score in participants with 22q11DS. Similarly, the overall *subjective appreciation index* (all conditions combined) was significantly associated with the ACIPS total score in participants with ASD.

*Social functioning* no significant correlation was observed between the *experiential index total scores* and SRS-2 and VABS-II socialization scores, neither overall nor in the social/non-social conditions. In participants with ASD, the overall *subjective appreciation index* (all conditions combined) was associated with the VABS-II socialization score. See Table 7 for statistical values.

**Table 7:** Correlation of future projection capacity (experiential index and subjective appreciation index) with anticipatory pleasure and social functioning

	TDs								
	Anticipatory pleasure (ACIPS)			Social functioning (SRS-2)			Social functioning (VABS-II)		
	Spearman correlation	Sig (2-tailed)	BH treshold	Spearman correlation	Sig (2-tailed)	BH treshold	Spearman correlation	Sig (2-tailed)	BH treshold
Experiential Index: difference scores									
Social	.050	.784	.005	− .124	.529	0.13	.154	.495	.016
Non-social	.105	.561	0.11	− .235	.228	.025	.070	.757	.008
Overall	.154	.392	.019	− .222	.256	.022	− .005	.981	.002
Global appreciation: difference scores									
Social	.171	.342	.025	− .115	.559	.013	− .044	.847	.011
Non-social	.010	.957	.002	− .119	.547	.016	.021	.925	.005
Overall	.156	.386	.022	− .126	.524	.019	− .038	.865	.008

	22q11DS								
	Anticipatory pleasure (ACIPS)			Social functioning (SRS-2)			Social functioning (VABS-II)		
	Spearman correlation	Sig (2-tailed)	BH treshold	Spearman correlation	Sig (2-tailed)	BH treshold	Spearman correlation	Sig (2-tailed)	BH treshold
Experiential index: difference scores									
Social	.205	.337	.022	.052	.815	.008	− 0.88	.690	.011
Non-social	− .007	.973	.005	− .226	.299	.025	.130	.555	.016
Overall	.169	.431	.019	− .106	.629	.013	− .007	.974	.002
Global appreciation: difference scores									
Social	.475	**.019**	.025	− .020	.928	.002	− .199	.362	.019
Non-social	.083	.698	.013	− .136	.537	.016	.063	.776	.011
Overall	.416	.043	.022	− .031	.887	.005	− .058	.793	.008

	ASD								
	Anticipatory pleasure (ACIPS)			Social functioning (SRS-2)			Social functioning (VABS-II)		
	Spearman correlation	Sig (2-tailed)	BH treshold	Spearman correlation	Sig (2-tailed)	BH treshold	Spearman correlation	Sig (2-tailed)	BH treshold
Experiential index: difference scores									
Social	.307	.201	.016	− .366	.149	.025	.246	.340	.008
Non-social	.256	.290	.011	− .293	.253	.013	.194	.456	.002
Overall	.310	.196	.019	− .350	.168	.022	.225	.386	.005
Global appreciation: difference scores									
Social	.529	.020	.019	− .267	.301	.002	.511	.036	.011
Non-social	.509	.026	.016	− .442	.075	.008	.523	.031	.013
Overall	.563	**.012**	.025	− .363	.152	.005	.564	**.018**	.022

Correlations sustaining Benjamini–Hochberg treshold are displayed in bold

Of note, all the analyses were run without the five participants with 22q11DS that scored higher than the clinical cutoff on the SCQ and the results remained unchanged.

## Discussion

The first aim of this study was to characterize EFT in two neurodevelopmental disorders, one already studied (ASD) and one in which EFT has never been studied (22q11DS). The second aim was to explore the links between EFT, AP and SF in these two conditions. Our main findings indicate that the narratives produced by individuals with ASD and 22q11DS were rated as less imaginable (*subjective appreciation index*) compared to TDs. Moreover, we found significant correlations between AP and the *subjective appreciation index* in the two groups and between SF and the *subjective appreciation index* only in participants with ASD. Despite comparable reductions in terms of vividness in individuals with 22q11DS and ASD, specific profiles emerged regarding the quality of the narratives: individuals with ASD told more categoric narratives than TDs, and 22q11DS individuals told less detailed narratives than TDs overall but also less than ASD individuals in the social conditions. Additionally, EFT was associated with VF in individuals with 22q11DS, whereas it was mostly associated with VIQ in participants with ASD.

### EFT and Associations with AP and SF

Narratives of both individuals with 22q11DS and ASD were rated as subjectively less vivid (i.e.* subjective appreciation index*) compared to those of TDs, regardless of the temporal or social conditions. In individuals with ASD, this finding replicates what has been shown in adults (Lind et al. 2014) and children with ASD (Ciaramelli et al. 2018), although the vividness was rated by the participants themselves in previous studies and not by the examiner. In individuals with 22q11DS, this is the first evidence of impairments in terms of vividness and imaginability of narratives. As this finding was not specific to a given condition (temporal or social), it might suggest the presence of a more general impairment in autonoetic consciousness—the ability to place oneself in time required in both EM and EFT (Buckner and Carroll 2007)—in both individuals with ASD and 22q11DS. Previous findings in the field of ASD are in line with this hypothesis, notably that fewer and less detailed autobiographical memories and reduced specificity in self-description (Tanweer et al. 2010) were reported in this population (Bruck et al. 2007; e.g., Crane and Goddard 2008). In 22q11DS, this is the first evidence of a reduced autonoetic consciousness, which therefore should be further explored in this population. Besides contributing to difficulties making memories and productions imaginable, reduced autonoetic consciousness was found to lead to general self-awareness impairments as well as difficulties in goal-directed and social behaviors (Gardiner 2001). This is supported by our findings, as we observed significant associations between the subjective appreciation index and SF in ASD individuals, regardless of the social or temporal conditions. To our knowledge, this is the first evidence that EFT is linked to SF in this population and gives support to the hypothesis that autonoetic consciousness is related to social behavior. Moreover, autonoetic consciousness impairments could contribute to the ASD phenotype (i.e. impairments in social interaction and communication) and therefore points towards new intervention targets. Furthermore, and contrary to our hypothesis, the social modality didn’t play a role in the EFT-SF association, which supports the idea that impaired autonoetic consciousness *overall* contributes to social impairments. Interestingly, EFT was not associated with SF in individuals with 22q11DS, suggesting that further studies are required in this domain.

Moreover, significant associations were found between the *subjective appreciation index* and AP in both clinical groups. An association between EFT and AP was previously reported in a sample of adults with depression (Hallford et al. 2020a), but this study used a combined score of details and vividness of the narratives, making it hard to distinguish between these two components. Moreover, only positive events were investigated in Hallford et al. (2020c), since it has been found that deficits in detail/vividness may be specific to positive events in depression (Holmes et al. 2016). As participants were not requested to only evoke positive events in the current study, further investigations are needed to explore the potential role of events’ valence on vividness. Finally, Hallford et al. (2020a) also assessed AP during the future thinking task, by asking participants how pleasurable is was to think about the experience they were telling, which wasn’t done in this study and could have given more information about the link between AP and EFT.

However, the present study is the first, to our knowledge, to find a link between AP and EFT in individuals with both ASD and 22q11DS, which supports the hypothesis that difficulties to anticipate pleasure partially rely on impaired EFT. Interestingly, the narratives of both groups differed from TDs on vividness, which was a subjective measure we administered by rating how imaginable the narrative appeared to the examiner.

### Distinctive Profiles Between Individuals with ASD and 22q11DS

Despite comparable reductions in terms of vividness in individuals with 22q11DS and ASD, specific profiles emerged regarding the quality of the narratives.

In individuals with ASD, a higher proportion of narratives was rated as *categoric*, both in the production and recollection conditions as well as in social and non-social contexts. However, there was no difference in the rate of *specific* narratives compared to TDs, contrary to what was reported in previous studies (e.g., Lind et al. 2014; Lind and Bowler 2010). It should be noted that these discrepancies could be related to scoring differences. Indeed, we classified narratives as *categoric* when the narratives contained no spatiotemporal indicators even if they *could* have fitted in a day, for instance “I would go to the grocery store”, whereas other studies may have classified this type of narrative as *specific*. From a qualitative point of view, the main reason why narratives were rated as *categoric* in the ASD group was that participants tended to list several tasks, activities or details instead of telling a story with a beginning and an end. Their answers often contained no spatio-temporal indicator and were therefore not precise enough to be classified as *specific* or even *extended*. The quality of these *categoric* recollections and productions could be interpreted in regard to the second criterion of autism, namely repetitive and restricted behaviors (RRB), that involves compulsions and rituals such as verbal routines. Incidentally, Terrett et al. (2013) suggested that difficulties in EFT in children with ASD could be explained by the core feature of inflexibility—another characteristic of the RRB criterion—that reduces the capacity of projecting oneself forward in time. Our results are in line with and expand this hypothesis to autobiographic EM, as individuals with ASD showed similar difficulties both in the recollection and production conditions. Interestingly, the *experiential index*, reflecting narratives’ richness (i.e. level of details), produced by ASD individuals was comparable to that of the control group in the social condition. Mixed findings were described regarding the number of details in previous studies (Anger et al. 2019; Ciaramelli et al. 2018; Lind et al. 2014; Terrett et al. 2013), which is potentially explained by the use of various methods to rate the number of details. For example, some studies (Ciaramelli et al. 2018; Terrett et al. 2013) distinguished between internal and external details, and others used information from different sources (e.g. questionnaire completed by the participants, information from the raters, etc. Lind et al. 2014) to create a general index. In the present study, the lack of significant difference between ASD individuals and TDs regarding the *experiential index* could rely on another characteristic of the ASD phenotype, namely sensory sensitivity. Indeed, unusual sensory processing, and particularly hyper-reactivity to sensory stimulation, is common among individuals with ASD (e.g., Crane et al. 2010), which could explain the high amount of details they gave. Additionally, Crane et al. (2009) found that individuals with ASD gave a higher proportion of sensory elements when narrating self-defining memories. To examine this hypothesis, we performed *post-hoc* analyses by subdividing the *experiential index* into sensory (olfactive/gustative, visual, auditive) and non-sensory details (emotions, actions, thoughts). Interestingly, ASD participants appeared to tell significantly less non-sensory details but as many sensory details than TDs. These results suggest that TDs followed the instructions and therefore provided both sensory and non-sensory details, whereas it was harder for participants with ASD to provide non-sensory details but equally easy to provide sensory details. As a general *experiential index* might prevent to detect qualitative differences related to the type of details, these *post-hoc* analyses highlight the need to subdivide this index into two subcomponents in further studies. Finally, our results point toward a role of verbal abilities (VIQ) in the production of details during the FTT task, which is similar to what has been described by Ciaramelli et al. (2018) and is consistent with the verbal nature of the task.

In individuals with 22q11DS, a higher proportion of overall narratives were also rated as *categoric*. From a qualitative point of view, the narratives were however very different from those of the ASD group. Indeed, participants with 22q11DS tended to tell very short narratives or repeated the target word without adding a lot of additional information or spatio-temporal indicators. In addition, a larger proportion of narratives in the *recollection* and *non-social* conditions were rated as *extended* in individuals with 22q11DS compared to TDs. Altogether, these results could be interpreted in light of impaired spatial and temporal perception reported in 22q11DS individuals (e.g., Simon 2008). Indeed, difficulties placing events within a specific time, making narratives either decontextualized (i.e. categoric) or too widely spread across time (i.e. extended), are observed in individuals with 22q11DS. Significant differences with the TD group were also found in the level of details contained in the narratives, as measured by the *experiential index*. Indeed, individuals with 22q11DS produced less detailed narratives than TDs (less sensory and non-sensory details contrary to the ASD group), especially in the social conditions. This could be interpreted in light of verbal initiation impairments, since we found significant associations between VF abilities and the experiential index only in this group of participants and specifically in the social conditions. Interestingly, previous studies have shown marked initiation impairments in 22q11DS (Maeder et al. 2016) and reported significant associations between initiation abilities and SF level (Dubourg et al. 2020). Altogether, these findings suggest that initiation difficulties—and potentially executive impairments more broadly—might contribute to social difficulties in this population. Future studies should further investigate the potential role of different executive functions on SF in this population. Moreover, no association was found with VIQ in this group, which suggests that different types of verbal abilities can play a role in EFT: verbal initiation (as measured by VF) in 22q11DS and broader verbal abilities (as measured by VIQ) in ASD.

### Strengths, Limitations, Future Directions and Clinical Implications

This is the very first study investigating EFT in 22q11DS, adding new information to the existing literature about this rare condition. Moreover, EFT was related with both AP and SF in individuals with ASD, adding important information to the existing body of evidence in this population. Finally, it is the first time that AP and SF are explored conjointly with EFT, as well as the distinction between social and non-social contexts.

However, the results of the present study should be interpreted in light of several methodological considerations. First of all, and given the verbal nature of the FTT, it could have been useful to include a control task aimed at assessing narrative skills, similar to what has been done in previous studies (Lind et al. 2014; Marini et al. 2019). Furthermore, the temporal distance of the future events (i.e. if the event would occur in the next 24 h/week/month/year etc.) was not controlled, contrary to what has been done in other studies (e.g Hallford et al. 2020a). Since temporal distance of future events has been shown to influence characteristics of EFT relevant to AP, including vividness and detail level (D’Argembeau and Van Der Linden 2004), this aspect should be investigated in future studies to specify the nature of the link between EFT and AP. Finally, a general measure of AP to distinguish whether associations with EFT were specific to social AP or AP in general would have been useful.

Secondly, heterogeneity of the clinical profiles in 22q11DS and in ASD should be considered when interpreting the results. In particular, five participants with 22q11DS scored above the clinical cutoff on the SCQ but the presence of an ASD diagnosis was not formally investigated in this subgroup. This represents an important distinction, as Angkustsiri et al. (2014) showed that individuals with 22q11DS can score above the clinical cutoff on the SCQ without meeting all diagnostic criteria for ASD. In their study, the SCQ and the ADOS were used to assess the presence of ASD in a sample of 29 children and adolescents and none of the participants had both SCQ and ADOS scores in the elevated range. Nevertheless, to investigate the impact of participants with an elevated SCQ score on the obtained results, all the analyses were conducted while excluding these five participants and the results remained unchanged. This suggests that the results obtained in the 22q11DS group are not explained by the presence of comorbid autistic traits in a subgroup of participants. Furthermore, even if we conducted a clinical interview to assess the presence of comorbid psychiatric conditions, our clinical samples were not large enough to make subgroups according to the presence of a specific diagnosis. It would be of particular interest to specifically investigate EFT in individuals with and without a comorbid mood disorder, since depression is known to be associated with impaired EFT. However, only 20% of individuals with ASD, and none of the participants in the 22q11DS group, met diagnostic criteria for a mood disorder*.* For this reason, we believe that the obtained results cannot be explained by psychiatric comorbidities only. In addition, anxiety disorders are the most frequent comorbidities observed in our clinical groups, but little is known about the role of anxiety in EFT. Future research should investigate the impact of anxio-depressive comorbidities in ASD and 22q11DS on EFT performances. Unfortunately, the potential impact of comorbidities, but also medications (50% of individuals with 22q11DS and 30% of individuals with ASD were under medications), remains unknown in the present study. Moreover, only relatively high functioning participants with ASD were included in this study because of the verbal nature of the task. Therefore, the present results cannot be extended to individuals with more impaired verbal and/or intellectual abilities. Finally, the sample size remains relatively small, but comparable to other studies on EFT (e.g., Anger et al. 2019; Ciaramelli et al. 2018; Lind et al. 2014).

As for clinical implications, some studies have shown that training EFT can lead to positive outcomes in healthy individuals (e.g., Brown et al. 2002; Schubert et al. 2020) but little is known in neurodevelopmental disorders. A recent study by Hallford et al. (2020c) trained EFT using the Episodic Future Thinking Test (EFT-T; Hallford et al. 2020b) and showed a significant improvement on the specificity, imaginary and level of detail of the narratives. Of particular interest, participants who received the intervention reported significantly higher scores on a questionnaire measuring AP. This kind of intervention could be particularly relevant for both individuals with 22q11DS and ASD, given their impairments in AP and SF. The use of visual cues could be particularly relevant in these populations, as Anger et al. (2019) showed that adding visual cues helped individuals with ASD performing as well as TDs during an episodic memory and future thinking task. Moreover, interventions targeting autonoetic conscientiousness could be implemented as the latest was found to be diminished in adults with ASD during an autobiographical memory task (e.g., Tanweer et al. 2010). Finally, and given the observed associations between verbal fluency and EFT in the 22q11DS sample, it would be of interest to explore the impact of a training focusing on verbal initiation.

## Conclusions

Overall, the results of the present study indicate EFT impairments in individuals with ASD and 22q11DS compared to TDs in terms of specificity, richness and vividness of the produced narratives. However, specific profiles of answers were found in each group, providing new information about each phenotype. During the task, participants had to tell narratives in social and non-social contexts, a distinction that has never been done before to our knowledge, and appear of a particular relevance in population characterized by impairments in social functioning. Overall, social context didn’t appear to make it more difficult to recall or produce an event, but, in 22q11DS, correlation with verbal initiation and its predictive role on SF seemed to be confirmed since they performed lower than ASD individuals in social contexts. Moreover, SF was found to be correlated with EFT in ASD individuals, which point toward potential interventions targeting autonoetic consciousness to improve SF. Finally, AP appeared to be correlated with EFT in both neurodevelopmental groups, showing for the first time the link between AP in EFT in these populations.

## Supplementary Information

Below is the link to the electronic supplementary material.

Supplementary file 1 (DOCX 14 KB)
