# Supplementary material for: Episodic Future Thinking in Autism Spectrum Disorder and 22q11.2 Deletion Syndrome: Association with Anticipatory Pleasure and Social Functioning
Source: J Autism Dev Disord. 2021 Feb 14;51(12):4587–604. doi: 10.1007/s10803-021-04903-2 (PMC8592949; doi:10.1007/s10803-021-04903-2)
Supplement: Supplementary file 1 — (DOCX 14 KB) [file 10803_2021_4903_MOESM1_ESM.docx]

**Narrative example**: recollection and social conditions

Yesterday, I went shopping at the supermarket of the Geneva train station with my colleague just before taking our train home. We ran into the store because we were in a hurry, in fact everyone seemed to be in a hurry, a lot of people were running around. We split up to buy everything we needed: I went to the vegetable department trying not to push too many people around. The avocados weren't ripe, which upset me, so I took a cucumber. Then I went past the bakery booth and it smelled good so I bought some bread, and afterwards I told myself that it was silly because I still had some at home. I also bought some vanilla soap, the bottle is yellow, and I almost confused it with the orange flower soap because of the yellowish bottle but I don't like the smell so I'm glad I didn't make that mistake. Then I joined my colleague, we stood in line and talked together. The cashier smiled at me and I thought she was very kind even though it was the end of the day and that she was probably fed up with all these stressed people. We ran off to catch our train, which luckily we got.

**Coding and rationale**:

*Narratives category*: specific 🡪 the event has a spatiotemporal indicator: it took place the day before, at the supermarket of the Geneva train station.

*Experiential index*: olfactive/gustative (smelled good, the smell; score 2), visual (a lot of, yellow, yellowish, ripe; score 2), auditive (none; score 0), emotions/feelings (upset, glad; score 2), actions (shop, run, split up, go, try, push, take, go, buy, buy, confuse, join, stand, run; score 2), thoughts (I told myself it was silly; I thought she was very kind; score 2) 🡪 total score = 10, sensory details score = 4, non-sensory details score = 6

*Subjective Appreciation index*: 4 (from 1 to 5) 🡪 the narrative was subjectively rated as 4 by the examiner, reflecting how vivid and imaginable it was for her

Note: stress doesn’t count as an emotion in this example because it is not the stress expressed by the participant telling the narrative but the stress attributed to other people involved in the story. The same reasoning was applied for the cashier smiling that doesn’t count as an action in this example.
